# Verses

**Published:** 1907-03

**Authors:** 


### VERSES.

### A MAN OF LETTERS.

Young Pillman's got a shingle out
Proclaiming him "M. D.,"
But from A. M. to late P. M.
His office is M. T.—*Exchange.*

### UNPREPARED.

A very thin girl named Miss Bratten,
Once went out to skate at Manhattan,
She soon struck the floor,
Said she: "Well! Before
I skate any more I must fatten."

—*Denver Post.*

### A PESSIMIST.

Spring is coming; what's the use?
'Tis shaking of the dice—
Slip on a banana peel
Instead of on the ice.

—*New York Sun.*

### "RIGHT OR WRONG."

Do good to all—by all be blessed;
Not ill for ill bestow;
To nature and to God the rest—
No good from bad can grow.—*Ex.*

### LIFE.

"Life is a mirror, for king or slave;
It is just what you are and do.
And the best will come back to you."—*Ex.*

### THE GROUND HOG.

De groun' hog cum out,
An' 'e look all eroun',
'E peer at de sky,
An' 'e gaze on de groun'.

W'en 'e seed no shadder,
'E frisk all erbout,
An' 'e say to 'e self,
I gwine ter stay out.

But all ob er sudden,
De clouds brake away;
De sun shine 'pon 'im,
An' 'e 'clude not ter stay.

So 'e skid ter 'e hole,
Ter sleep 'dout honger,
Till winter hold out
Fer six weeks longer.

—B. H. Teague.

